# A Case Report of Non-Bacterial Cystitis Caused by Immune Checkpoint Inhibitors

**DOI:** 10.3389/fimmu.2021.788629

**Published:** 2021-12-23

**Authors:** Sihui Zhu, Lijuan Bian, Jia Lv, Baorui Liu, Jie Shen

**Affiliations:** ^1^ Comprehensive Cancer Centre of Nanjing Drum Tower Hospital, Clinical College of Nanjing Medical University, Nanjing, China; ^2^ Comprehensive Cancer Centre of Drum Tower Hospital, Medical School of Nanjing University, Clinical Cancer Institute of Nanjing University, Nanjing, China

**Keywords:** programmed death-1 antibody, programmed cell death-ligand 1 antibody, intrahepatic cholangiocarcinoma, cystitis, immune-related adverse events

## Abstract

We report a case of non-bacterial cystitis after treatment with programmed death-1 (PD-1) and programmed cell death-ligand 1 (PD-L1) antibodies, which was considered an immune-related adverse event (irAE). A 48-year-old male patient with intrahepatic cholangiocarcinoma (ICC) was treated with nivolumab after postoperative multi-line treatment. This patient recurred worsening of psoriasis and repeated urinary tract discomfort. The drug was discontinued and surgery was performed due to the recurrence of the tumor suggested by imaging. After receiving three cycles of chemotherapy treatment combined with atezolizumab, urinary tract discomfort reappeared. No bacteria were found in multiple urine cultures, and non-bacterial bladder inflammation was considered after cystoscopy biopsy. This is a report of non-bacterial inflammation of the urinary tract caused by immunotherapy.

## Background

Immune checkpoint inhibitors (ICIs) induce antitumor immune responses by blocking immune checkpoints, such as cytotoxic T lymphocyte antigen-4 (CTLA-4) and programmed death-1 (PD-1) or its ligand, PD-L1. By increasing the activity of the immune system, these drugs produce excessive immunity to normal organs and cause toxicity different from standard chemotherapy or other biological agents (1). Among them, skin and gastrointestinal adverse reactions were the most commonly observed (2). Here, we reporte a case of cystitis after receiving nivolumab and atezolizumab (PD-1 and PD-L1 antibodies), which was considered a type of immune-related adverse event (irAE).

## Case Report

**Figure 1:**
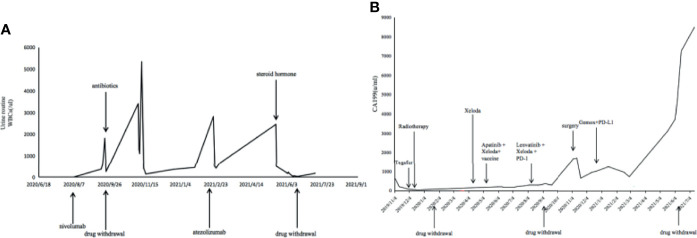
**(A)** Clinical course. **(B)** Carbohydrate antigen 19-9 (CA19-9) change curve.

**Figure 2:**
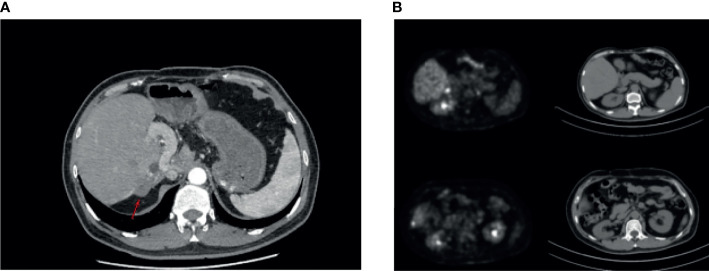
**(A)** October 22, 2019: magnetic resonance cholangiopancreatography showed the density of the soft tissue under the capsule of the right lobe of the liver. The possibility of metastasis was considered. **(B)** November 4, 2020: discovery of new lesions; tumor metastasis was considered (PET/CT showed nodular thickening of the capsule of the right posterior lobe of the liver, soft tissue density nodules in the fat space above the duodenum on the right side of pancreatic head in the abdominal cavity, and abnormal increase of glucose metabolism). Compared with the previous film, it was a new lesion. A high possibility of tumor metastasis was considered.

**Figure 3:**
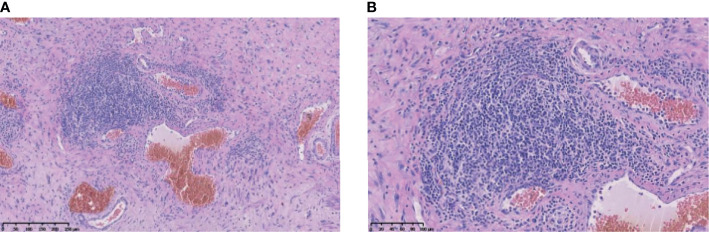
Histopathological findings. **(A)** was taken at 10x, and **(B)** was taken at 20x.

Our patient is a 48-year-old male with intrahepatic cholangiocarcinoma (ICC) with a history of psoriasis for more than 20 years. After receiving multi-line treatment, including surgery, chemotherapy, and radiotherapy, among others, the effect was not good and the patient’s disease progressed. According to the results of clinical trials on biliary tumors (3), PD-1 antibody combined with lenvatinib was tried. There was no obvious abnormality in urine routine during baseline examination; meanwhile, the patient’s psoriasis was also in an inactive period, which has been stable for a long time and did not require drug control. However, after combination treatment with lenvatinib and nivolumab, psoriasis recurred, with scattered rashes all over the body, and skin toxicity symptoms aggravated with the increase of the cumulative dose of the PD-1 antibody. The patient developed urinary tract irritation after three cycles. Urine examination revealed 375/μl white blood cells (WBCs) in the urine (**Figure 1**). Although no bacteria were found in the urine culture, the possibility of bacterial cystitis was still considered after consultation with the urology department based on the patient’s symptoms at that time. After antibiotic treatment, laboratory tests had indeed improved, but the patient’s symptoms did not get better. Therefore, the treatment was discontinued. However, the patient’s tumor indicators increased due to the discontinuation of lenvatinib and PD-1 antibody (**Figure 1**). Imaging examinations also revealed new lesions located on the right posterior lobe of the liver and above the duodenum on the right side of the pancreatic head in the abdominal cavity (**Figure 2**). In general surgery consultation, this patient was considered to have a chance of surgery, so an operation was performed. One month after the second operation, the patient’s tumor indicators decreased and then increased again (**Figure 1**). Except for a slight thickening of the local omentum, no lesions were found on imaging. At the same time, the patient’s urinary tract symptoms improved after the drug has been withdrawn for more than 3 months. Therefore, Gemox chemotherapy (gemcitabine+oxaliplatin) was considered after the operation. Meanwhile, the patient’s immunohistochemistry was positive for PD-L1. Taking the improvement of curative effect into account, and with the patient’s insistence on trying, the treatment plan was finally determined as Gemox combined with the PD-L1 antibody. The patient then did not develop skin toxicity. After three cycles, the tumor markers dropped significantly, but bladder irritation reoccurred, which was significantly worse than before. Urine examination revealed that WBCs were 2,818/μl (**Figure 1**) and bacteria were 512/μl. After consultation with the Department of Urology and Nephrology, bilateral ureteral stent implantation and cystoscopy and biopsy were decided. The result of the bladder biopsy indicated chronic inflammation of mucosal tissue, mucosal erosion in some areas, and proliferation of granulation tissues and fibroblasts (**Figure 3**). After the multidisciplinary consultation, immune-related cystitis was considered; treatment with steroid hormones was given, which started at 2 mg/kg, then slowly decreased. The urinary tract irritation symptoms were relieved, and the laboratory examination was also significantly improved. After 4 weeks, the urine routine was reviewed, and the WBC count was 66/μl. At present, during the hormone reduction period, urine routine and carbohydrate antigen 19-9 (CA19-9) are continued to be monitored, and imaging examinations are performed regularly (including MRI and PET-CT). In the follow-up treatment, it was considered that this patient cannot tolerate the side effects of immunotherapy. Whether hormones affect the efficacy of immunotherapy is currently controversial, so chemotherapy only was considered.

## Discussion

We report a case of non-bacterial cystitis that is related to PD-1 and PD-L1 antibodies after treatment. As far as we know, there are currently few reports of irAEs in the urinary system, and non-bacterial cystitis is even rarer. This adverse reaction usually causes bladder irritation symptoms, such as frequent urination and pain on urination. For example, a case of immune-related cystitis after the use of nivolumab has been reported by Ozaki et al. This patient developed frequent urination, painful urination, and gross hematuria (4). In addition, two cases reported by Shimatani et al. had symptoms such as frequent urination and diarrhea, and the final pathological examination results were clear as immune-related cystitis (5). According to the relevant literature and guidelines, once irAE is suspected, it is best to have a specialist consultation to rule out infection or other accidental conditions (4). Management after diagnosis generally relies on steroid hormones and other immunomodulatory agents (6).

The exact pathophysiological mechanism of irAE is still unclear, and the side effects and severity caused by different ICI drugs or even different doses also vary (1). In an analysis of advanced melanoma, 71% of patients treated with nivolumab had irAEs, the most common of which were fatigue, pruritus, diarrhea, and rash. Of the patients, 10% reached grade 3 or 4 (7). On the other hand, 84.6% of patients treated with ipilimumab had irAEs and 25.2% of patients reached grade 3 or 4, which mainly occurred in the gastrointestinal tract, liver, skin, and the endocrine system (7). As far as we know, the non-bacterial cystitis that we have reported in the urinary system is relatively rare, and there are very few reports. Multiple studies have shown that patients with a history of autoimmune diseases have an increased risk of irAEs (1, 8). But these adverse reactions can often be controlled by active treatment, so this is not an absolute contraindication for ICI use. Such patients can still obtain long-lasting antitumor effects from immunotherapy (9).

Steroid hormones can regulate immune activity by inducing the apoptosis of T lymphocytes (10), but its influence on ICI therapy is still controversial. Horvat et al. found that one-third of patients receiving ipilimumab required systemic corticosteroid therapy. But it did not affect the efficacy of immunotherapy. They found that receiving corticosteroid therapy had no significant relationship with the patients’ overall survival (OS) (11). However, there are some studies suggesting that a high baseline prednisone dose is associated with poor survival (12). They speculated that the use of steroid hormones before ICI treatment may affect the activation of primitive T cells, thereby affecting the efficacy of immunotherapy, while the use of steroid hormones during treatment would only affect the activated T cells, and this effect cannot reduce the overall efficacy of immunotherapy (12). In this case, the patient had a history of psoriasis. After using PD-1, psoriasis worsened and the symptoms were relieved after symptomatic treatment, so his underlying disease did not affect the ICI treatment. After three cycles, the patient developed symptoms of bladder irritation, but because of the unsatisfactory treatment effect, the disease progression was found on imaging examination to be the reason for the patient’s discontinuation of the PD-1 antibody. In the subsequent PD-L1 antibody treatment, the patient did not develop skin toxicity, but the bladder symptoms continued and were worse than before. It may be that the signal pathways of PD-1 and PD-L1 are not the same and that the mechanisms of adverse reactions are also different. After multidisciplinary consultation, the bladder biopsy was considered to be non-bacterial cystitis caused by immunotherapy. After hormone therapy, the patient’s own symptoms and laboratory examinations were significantly improved. Since the patient complained that he cannot tolerate such symptoms, and whether hormone therapy would affect the efficacy of ICI is currently controversial, he did not continue using immunotherapy.

According to statistics, globally, the probability of death due to irAEs that received ICI treatment is about 0.6%. Of this, PD-1 antibody contributes 0.36% and PD-L1 0.38% (13). The cause of death is usually pneumonia, hepatitis, or neurotoxicity. CTLA-4 and combined immunotherapy have higher mortality rates, 1.08% and 1.23%, respectively (13). Studies have found that certain antibodies in the serum before treatment may be related to the risk of irAEs (9). An early increase in peripheral lymphocyte counts can also be used as a biomarker of irAE risk (14). B cells in the body and some cytokines such as interleukin 17 (IL-17) can also help identify the risk of irAE in patients (15, 16), but they appear to lack sensitivity. Further research is needed in the future (1, 9).

More and more studies have shown that the onset of irAE can be used as a potential biomarker to predict the efficacy of PD-1 and PD-L1 in various solid tumors (17). Patients with irAEs show better efficacy than those without irAE (14, 18, 19). The mechanism is unclear, but may be due to the bystander effect of ICI-activated T cells, which target not only tumor cells but also normal tissues to produce side effects (20). The specific antigens preexisting in normal organs may also be the predisposing factors of irAEs and produce autoimmune toxic reactions that are not antitumor related (21). Of course, this conclusion also needs the support of more clinical data.

## Data Availability Statement

The raw data supporting the conclusions of this article will be made available by the authors, without undue reservation.

## Ethics Statement

The studies involving human participants were reviewed and approved by the Ethics Committee of the Comprehensive Cancer Center of Drum Tower Hospital of Nanjing University. The patients/participants provided written informed consent to participate in this study. Written informed consent was obtained from the individual(s) for the publication of any potentially identifiable images or data included in this article.

## Author Contributions

JS and BL designed the clinical trial. SZ, JS, and BL drafted the manuscript and provided figures. SZ, LB, JL, BL, and JS acquired, analyzed, and interpreted the data. JS, LB, and BL revised the manuscript critically for important intellectual content and agreed to be accountable for all aspects of the work in ensuring that questions related to the accuracy or integrity of any part of the work are appropriately investigated and resolved. All authors contributed to the article and approved the submitted version.

## Funding

This study was supported by the National Natural Science Foundation of China (no. 81902914), Jiangsu Provincial Medical Youth Talent (no. QNRC2016043), and the Key Medical Science and Technology Development Project of Nanjing (no. ZKX16032).

## Conflict of Interest

The authors declare that the research was conducted in the absence of any commercial or financial relationships that could be construed as a potential conflict of interest.

## Publisher’s Note

All claims expressed in this article are solely those of the authors and do not necessarily represent those of their affiliated organizations, or those of the publisher, the editors and the reviewers. Any product that may be evaluated in this article, or claim that may be made by its manufacturer, is not guaranteed or endorsed by the publisher.

## References

[1] Postow M Sidlow R Hellmann M. Immune-Related Adverse Events Associated With Immune Checkpoint Blockade. N Engl J Med (2018) 378:158–68. doi: 10.1056/NEJMra1703481 29320654

[2] Weber J Hodi F Wolchok J Topalian S Schadendorf D Larkin J. Safety Profile of Nivolumab Monotherapy: A Pooled Analysis of Patients With Advanced Melanoma. J Clin Oncol (2017) 35:785–92. doi: 10.1200/JCO.2015.66.1389 28068177

[3] Lin J Yang X Long J Zhao S Mao J Wang D. Pembrolizumab Combined With Lenvatinib as Non-First-Line Therapy in Patients With Refractory Biliary Tract Carcinoma. Hepatobiliary Surg Nutr (2020) 9:414–24. doi: 10.21037/hbsn-20-338 PMC7423565 32832493

[4] Ozaki K Takahashi H Murakami Y Kiyoku H Kanayama H. A Case of Cystitis After Administration of Nivolumab. Int Cancer Conf J (2017) 6:164–6. doi: 10.1007/s13691-017-0298-6 PMC6498356 31149494

[5] Shimatani K Yoshimoto T Doi Y Sonoda T Yamamoto S Kanematsu A. Two Cases of Nonbacterial Cystitis Associated With Nivolumab, the Anti-Programmed-Death-Receptor-1 Inhibitor. Urol Case Rep (2018) 17:97–9. doi: 10.1016/j.eucr.2017.12.006 PMC5849865 29541592

[6] Puzanov I Diab A Abdallah K Bingham C Brogdon C Dadu R. Managing Toxicities Associated With Immune Checkpoint Inhibitors: Consensus Recommendations From the Society for Immunotherapy of Cancer (SITC) Toxicity Management Working Group. J Immunother Cancer (2017) 5:95. doi: 10.1186/s40425-017-0300-z 29162153 PMC5697162

[7] Weber J Kähler K Hauschild A. Management of Immune-Related Adverse Events and Kinetics of Response With Ipilimumab. J Clin Oncol (2012) 30:2691–7. doi: 10.1200/JCO.2012.41.6750 22614989

[8] Abdel-Wahab N Shah M Lopez-Olivo M Suarez-Almazor M. Use of Immune Checkpoint Inhibitors in the Treatment of Patients With Cancer and Preexisting Autoimmune Disease: A Systematic Review. Ann Internal Med (2018) 168:121–30. doi: 10.7326/M17-2073 29297009

[9] Yoest J. Clinical Features, Predictive Correlates, and Pathophysiology of Immune-Related Adverse Events in Immune Checkpoint Inhibitor Treatments in Cancer: A Short Review. ImmunoTargets Ther (2017) 6:73–82. doi: 10.2147/ITT.S126227 29067284 PMC5644546

[10] Herold M McPherson K Reichardt H. Glucocorticoids in T Cell Apoptosis and Function. Cell Mol Life Sci CMLS (2006) 63:60–72. doi: 10.1007/s00018-005-5390-y 16314919 PMC2792342

[11] Horvat T Adel N Dang T Momtaz P Postow M Callahan M. Immune-Related Adverse Events, Need for Systemic Immunosuppression, and Effects on Survival and Time to Treatment Failure in Patients With Melanoma Treated With Ipilimumab at Memorial Sloan Kettering Cancer Center. J Clin Oncol (2015) 33:3193–8. doi: 10.1200/JCO.2015.60.8448 PMC5087335 26282644

[12] Kartolo A Deluce J Holstead R Hopman W Lenehan J Baetz T. Impact of Baseline Corticosteroids on Immunotherapy Efficacy in Patients With Advanced Melanoma. J Immunother (Hagerstown Md 1997) (2021) 44:167–74. doi: 10.1097/CJI.0000000000000360 33560702

[13] Wang D Salem J Cohen J Chandra S Menzer C Ye F. Fatal Toxic Effects Associated With Immune Checkpoint Inhibitors: A Systematic Review and Meta-Analysis. JAMA Oncol (2018) 4:1721–8. doi: 10.1001/jamaoncol.2018.3923 PMC6440712 30242316

[14] Okada N Kawazoe H Takechi K Matsudate Y Utsunomiya R Zamami Y. Association Between Immune-Related Adverse Events and Clinical Efficacy in Patients With Melanoma Treated With Nivolumab: A Multicenter Retrospective Study. Clin Ther (2019) 41:59–67. doi: 10.1016/j.clinthera.2018.11.004 30528047

[15] Das R Bar N Ferreira M Newman A Zhang L Bailur J. Early B Cell Changes Predict Autoimmunity Following Combination Immune Checkpoint Blockade. J Clin Invest (2018) 128:715–20. doi: 10.1172/JCI96798 PMC5785243 29309048

[16] Young A Quandt Z Bluestone J. The Balancing Act Between Cancer Immunity and Autoimmunity in Response to Immunotherapy. Cancer Immunol Res (2018) 6:1445–52. doi: 10.1158/2326-6066.CIR-18-0487 PMC6281171 30510057

[17] Das S Johnson D. Immune-Related Adverse Events and Anti-Tumor Efficacy of Immune Checkpoint Inhibitors. J Immunother Cancer (2019) 7:306. doi: 10.1186/s40425-019-0805-8 31730012 PMC6858629

[18] Rogado J Sánchez-Torres J Romero-Laorden N Ballesteros A Pacheco-Barcia V Ramos-Leví A. Immune-Related Adverse Events Predict the Therapeutic Efficacy of Anti-PD-1 Antibodies in Cancer Patients. Eur J Cancer (Oxford Engl 1990) (2019) 109:21–7. doi: 10.1016/j.ejca.2018.10.014 30682533

[19] Ricciuti B Genova C De Giglio A Bassanelli M Dal Bello M Metroí G. Impact of Immune-Related Adverse Events on Survival in Patients With Advanced Non-Small Cell Lung Cancer Treated With Nivolumab: Long-Term Outcomes From a Multi-Institutional Analysis. J Cancer Res Clin Oncol (2019) 145:479–85. doi: 10.1007/s00432-018-2805-3 PMC11810236 30506406

[20] Passat T Touchefeu Y Gervois N Jarry A Bossard C Bennouna J. [Physiopathological Mechanisms of Immune-Related Adverse Events Induced by Anti-CTLA-4, Anti-PD-1 and Anti-PD-L1 Antibodies in Cancer Treatment]. Bull du Cancer (2018) 105:1033–41. doi: 10.1016/j.bulcan.2018.07.005 30244981

[21] Iwama S De Remigis A Callahan M Slovin S Wolchok J Caturegli P. Pituitary Expression of CTLA-4 Mediates Hypophysitis Secondary to Administration of CTLA-4 Blocking Antibody. Sci Trans Med (2014) 6:230ra45. doi: 10.1126/scitranslmed.3008002 24695685

